# Plain versus drug balloon and stenting in severe ischaemia of the leg (BASIL-3): open label, three arm, randomised, multicentre, phase 3 trial

**DOI:** 10.1136/bmj-2024-080881

**Published:** 2025-02-24

**Authors:** Andrew W Bradbury, Jack A Hall, Matthew A Popplewell, Lewis Meecham, Gareth R Bate, Lisa Kelly, Jon J Deeks, Catherine A Moakes, BASIL-3 Investigators, Arul Ganeshan, Hany Zayed, Robert Davies, Ian Chetter, Stephen Butterfield, Stephen Goode, James Metcalfe, Peter Mezes, Simon Hobbs, Nimit Goyal, Jai Patel, Mario Caruana, Tawqeer Rashid, Neelan Das, Nityanand Arya, Patrick Chong, Said Habib, Richard White, George Antoniou, Alun Davies, Angela Rogers, Shiva Dindyal, Nikolas Arestis, Graham Weir, Philip Davey, Raj Das, Gerard Stansby, Ron Eifell, Phillip Davey, John Asquith, Lasantha Wijesinghe, Arndam Chaudhuri, Joseph Sathianathan, Nagendra Thayur, Farhan Ahmad

**Affiliations:** 1 University of Birmingham, Birmingham, UK; 2 Birmingham Clinical Trials Unit, School of Health Sciences, University of Birmingham, Birmingham, UK; 3 Black Country Vascular Network, Dudley, UK; 4 Department of Applied Health Sciences, University of Birmingham, Birmingham, UK; 5 University Hospital of Wales, Cardiff, UK; 6 University Hospitals Birmingham NHS Foundation Trust, Birmingham, UK; 7 NIHR Birmingham Biomedical Research Centre, Birmingham, UK

## Abstract

**Objective:**

To determine which primary endovascular revascularisation strategy represents the most clinically effective treatment for patients with chronic limb threatening ischaemia who require endovascular femoro-popliteal, with or without infra-popliteal, revascularisation.

**Design:**

Three arm, open label, pragmatic, multicentre, randomised, phase 3 superiority trial (BASIL-3).

**Setting:**

35 UK NHS vascular units.

**Participants:**

Patients with chronic limb threatening ischaemia who required endovascular femoro-popliteal, with or without infra-popliteal, revascularisation.

**Interventions:**

Participants were randomly assigned (1:1:1) to femoro-popliteal plain balloon angioplasty with or without bare metal stenting (PBA±BMS), drug coated balloon angioplasty with or without bare metal stenting (DCBA±BMS), or drug eluting stenting (DES) as their first revascularisation strategy.

**Main outcome measures:**

The primary outcome was amputation free survival defined as time to first major amputation or death from any cause. Secondary outcomes included the composite components of the primary outcome, major adverse limb events, major adverse cardiac events, and other prespecified clinical and patient reported outcome measures. Serious adverse events were collected up to 30 days after the first revascularisation procedure.

**Results:**

Between 29 January 2016 and 31 August 2021, 481 participants were randomised (167 (35%) women, mean age 71.8 years (standard deviation 10.8)). Major amputation or death occurred in 106 of 160 (66%) participants in the PBA±BMS group, 97 of 161 (60%) in the DCBA±BMS group, and 93 of 159 (58%) in the DES group (adjusted hazard ratios: PBA±BMS *v* DCBA±BMS: 0.84, 97.5% confidence interval 0.61 to 1.16, P=0.22; PBA±BMS *v* DES: 0.83, 0.60 to 1.15, P=0.20). No differences in serious adverse events were reported between the groups.

**Conclusions:**

Neither DCBA±BMS nor DES conferred significant clinical benefit over PBA±BMS in the femoro-popliteal segment in patients with chronic limb threatening ischaemia undergoing endovascular femoro-popliteal, with or without infra-popliteal, revascularisation.

**Trial registration:**

ISRCTN registry ISRCTN14469736

## Introduction

Chronic limb threatening ischaemia is the severest form of atherosclerotic peripheral arterial disease and presents with ischaemic pain with or without tissue loss. This disease represents a growing global healthcare burden, mainly owing to tobacco smoking, diabetes mellitus, and ageing populations.^1^ Unless perfusion is restored, patients are at high risk of major amputation and death, and so virtually all patients should be considered for revascularisation.

To date, three published, publicly funded randomised controlled trials have compared the clinical and cost effectiveness of different revascularisation strategies in chronic limb threatening ischaemia. The UK bypass versus angioplasty in severe ischaemia of the leg (BASIL) trial suggested that patients with mostly femoropopliteal disease and a life expectancy greater than two years should be offered surgical bypass in preference to plain balloon angioplasty (PBA).^2^ The United States best endovascular versus best surgical therapy in patients with chronic limb threatening ischaemia (BEST-CLI) trial reported that in patients with an optimal vein for vein bypass (cohort one), major adverse limb events or death were lower in the vein bypass group than in those receiving endovascular treatment for similar disease. In a second cohort of patients without an optimal vein, outcomes were similar.^3^ The UK BASIL-2 trial reported that, in patients requiring an infra-popliteal, with or without a femoro-popliteal, revascularisation, a best endovascular treatment strategy resulted in better amputation free survival than vein bypass, mainly because of more deaths throughout follow-up after vein bypass.^4^

However, in patients with chronic limb threatening ischaemia selected for endovascular revascularisation in preference to surgical revascularisation, considerable uncertainty exists about the relative effectiveness of different procedures, and in particular, the role of PBA with or without bare metal stenting (BMS), drug coated balloon angioplasty (DCBA) with or without BMS, and drug eluting stenting (DES). This lack of evidence is reflected in systematic reviews, meta-analyses, and published guidelines.^1^ ^5^ In 2012, the UK National Institute for Health and Care Excellence (NICE) recommended a randomised controlled trial to compare the clinical and cost effectiveness of PBA, BMS, DCBA, and DES in patients with chronic limb threatening ischaemia.^6^ This led the UK National Institute for Health and Care Research, Health Technology Assessment (NIHR HTA) programme to fund the balloon versus stenting in severe ischaemia of the leg—3 (BASIL-3) randomised controlled trial reported here. The aim of this trial was to determine which procedure resulted in better amputation free survival in patients with chronic limb threatening ischaemia who required endovascular femoro-popliteal, with or without infra-popliteal, revascularisation. Three strategies were compared: femoro-popliteal PBA±BMS, DCBA±BMS, or primary DES as the first revascularisation strategy. The BASIL-3 health economic analysis will be reported separately.

## Materials and methods

### Study design and participants

BASIL-3 was an open label, pragmatic, three arm, multicentre, superiority, phase 3, randomised controlled trial conducted at most (35) of the major UK NHS vascular units.^7^ Eligible participants were those who presented with chronic limb threatening ischaemia caused by atherosclerotic peripheral arterial disease and who, after a process of shared decision making, were offered and consented to a femoro-popliteal, with or without an infra-popliteal, endovascular revascularisation in preference to surgical revascularisation. As is standard UK practice, chronic limb threatening ischaemia was diagnosed on the basis of history and clinical examination, supported by selective use of haemodynamic testing and arterial imaging (one or more of duplex ultrasound, computed tomography angiography, magnetic resonance angiography, and digital subtraction angiography). To be randomised, a patient had to be assessed by a multidisciplinary team (including at least two vascular surgery or interventional radiology consultants), and deemed suitable for all three endovascular strategies. None of the patients randomised had undergone previous major (above the ankle) amputation of the trial leg. Patients presenting with intermittent claudication were not eligible for enrolment. None of the participants had a planned major (above ankle) amputation of the trial leg at the point of randomisation. Patients were excluded if they were expected to live for less than six months (pragmatic decision by randomising team) or had undergone an intervention to the target femoro-popliteal vessel within the past 12 months. Participants had to be able and willing to complete the health related quality of life and health economic questionnaires. Additionally, they needed to speak sufficient English (where translation facilities were insufficient) and to have capacity to provide written informed consent. The National Research Ethics Committee, North of Scotland, provided ethical approval on 26 August 2015 (15/NS/0070). Declaration of Helsinki and Good Clinical Practice guidelines were followed.

### Randomisation and masking

Participants were randomly assigned (1:1:1) using a secure online system to femoro-popliteal PBA±BMS, DCBA±BMS, or DES as their first revascularisation procedure. Minimisation was used to balance assignments according to age (≤60 years, 61-70 years, 71-80 years, >80 years), sex (male, female), diabetes mellitus (yes, no), chronic kidney disease (yes, no), severity of clinical disease (ischaemic rest or night pain only, tissue loss only, both), previous (permissible) intervention to the trial leg (yes, no), target artery (superficial femoral artery only, popliteal artery only, both), intention for a hybrid (endovascular with additional surgical) procedure (yes, no), and recruiting centre. Randomisation was provided centrally by the Birmingham Clinical Trials Unit, University of Birmingham, UK. Participants, study staff, and investigators were not masked to treatment allocation.

### Procedures

Vascular surgeons and interventional radiologists were encouraged to perform the allocated endovascular procedures using their preferred techniques and devices. Any UK licensed PBA, DCBA, BMS, or DES was permitted. The allocated intervention was to be performed within two weeks of randomisation where possible and clinically appropriate. All additional management strategies and procedures, including wound care and medical therapy, were at the discretion of the responsible clinicians and in the best interests of the patient. Participants were followed locally one month after the initial revascularisation; six, 12, and 24 months after randomisation; and then annually until the last participant had been followed for 24 months. Clinical (haemodynamic, medical treatments, clinical status) and health related quality of life data were collected during these visits. When face-to-face visits were not possible (particularly during covid-19), as much data as possible were obtained by telephone. In England and Wales, death and major amputation data were obtained until the end of follow-up from NHS Digital (the statutory custodian for health and social care data for England and Wales).

### Outcomes

The primary outcome was amputation free survival, defined as the time to major (above ankle) amputation of the trial leg or death from any cause (whichever occurred first, time-to-event analysis). Clinical secondary outcomes included time to death from any cause (overall survival); time to major amputation of the trial leg; further major revascularisation of the trial leg; major adverse limb events (defined as major amputation of the trial leg or additional major revascularisation of the trial leg); major adverse cardiovascular events (defined as a new chronic limb threatening ischaemia affecting the non-trial leg, major amputation of the non-trial leg, myocardial infarction, stroke, or transient ischaemic attack); 30 day (after first intervention) mortality and morbidity; relief of ischaemic pain (assessed using the visual analogue scale, the Vascular Quality of Life Questionnaire tool and opiate usage); healing of tissue loss (assessed using the perfusion, extent, depth, infection, and sensation (PEDIS), and wound, ischaemic, and foot infection (WIFi) scoring systems); and changes in ankle brachial pressure index and/or toe brachial pressure index. Health related quality of life was assessed using generic (Euroqol 5D 5L, Short Form-12, ICEpop capability measure for older people, and Hospital Anxiety and Depression Scale) and disease specific (Vascular Quality of Life Questionnaire) tools. Serious adverse events were recorded up to 30 days after the first post-randomisation revascularisation. Participants were defined as adherent to the allocated trial procedure if the first revascularisation after randomisation was endovascular, the randomised class of device was used, and the randomised device was used in the superficial femoral artery, in the popliteal artery, or in both.

### Statistical analysis

The original sample size was based on a time-to-event analysis making two key comparisons: PBA±BMS *v* DES, and PBA±BMS *v* DCBA±BMS. Recruitment was to take place over three years (20% year 1, 40% in years 2 and 3) with a minimum follow-up of two years in all participants. Based on the BASIL-1 trial, amputation free survival rates were assumed to be 0.70 in year 1, 0.64 in year 2, 0.52 in year 3, 0.46 in year 4, and 0.36 in year 5.^8^ Allowing for 5% attrition and the BASIL-1 survival estimates, 861 participants (having 342 primary outcome events) would provide 90% power to detect a reduction in amputation free survival of 40% (hazard ratio 0.60, equivalent to an absolute difference in amputation free survival of 13% at year 2, corresponding to one out of seven participants needing to benefit from DES or DCBA over PBA (number needed to treat) at the 2.5% significance level (to account for increased type I error risk associated with making two key comparisons) using the artsurv (version 1.0.7) programme in Stata (version 17.0). However, an error was identified in the implementation of the macro used to compute the original sample size, whereby a smaller effect size (than the targeted 40% relative reduction) had been applied for one of the two comparisons. This resulted in an overestimation of the sample size and number of primary outcome events needed. Using the same parameters as in the original calculation (listed above), the corrected sample size was 749 and the number of primary outcome events required was 291.

Because the anticipated recruitment rates were not achieved, recruitment continued beyond year 3, and the median follow-up was longer than planned. Therefore, the number of randomised patients required to observe the target number of 291 events for 90% power was reduced. With support of the funder and independent oversight from the data monitoring committee, recruitment rates, length of follow-up, and pooled event rates over time were modelled to predict the number of participants needed to reach 291 events, with a minimum follow-up of two years. Modelling was updated approximately every six months based on emerging data. BASIL-3 closed to recruitment on 31 August 2021, with 481 participants randomised and 296 primary outcomes observed.

A statistical analysis plan was specified before the analysis. All outcomes were analysed in the intention-to-treat population (all randomised participants irrespective of adherence). Differences between groups were presented with two sided 97.5% confidence intervals, adjusted for minimisation variables as fixed effects, and recruiting centre as a random effect (or as a shared frailty variable in the time-to-event analyses) when convergence was possible. P values are only presented for the primary outcome and were not corrected for multiple comparisons.

Amputation free survival was analysed using a Cox proportional hazards model to generate an adjusted hazard ratio. Statistical significance of the treatment group parameter was determined through examination of the associated χ^2^ statistic. Kaplan-Meier survival curves were constructed for visual presentation and absolute differences and number needed to treat values in failure probabilities (with 97.5% confidence intervals) were estimated between groups from these curves, computed at two years (as in the justification for the study sample size) and five years (the official final follow-up point). The confidence intervals for number needed to treat are presented in the notation of number needed to treat for benefit (NNTB) and for harm (NNTH) as introduced by Altman.^9^ A further prespecified analysis was conducted using a flexible parametric model with a time varying covariate for treatment to consider the effects of non-proportional hazards. The number of internal knots for the baseline hazard function and time varying covariate was determined from the model with the lowest Bayesian information criterion. A plot of hazard ratio over time (with corresponding 97.5% confidence intervals) was estimated.

Overall survival was analysed as per the primary outcome (amputation free survival). Other secondary time-to-event outcomes (major amputations, major adverse limb events, major adverse cardiovascular events) were considered in a competing risks framework to account for participants who died before reporting an event. Cause specific hazard ratios and subdistribution hazard ratios were estimated using cause specific Cox models and Fine-Grey models, respectively.^10^ ^11^ Cumulative incidence plots were produced for visual presentation.

Continuous secondary outcomes were summarised as means and standard deviations at each time point where appropriate, and adjusted mean differences were estimated using mixed effects repeated measures linear regression models. Binary secondary outcomes were summarised as rates and frequencies at each time point if appropriate. Adjusted risk ratios or adjusted risk differences were estimated using log binomial/identity binomial generalised linear mixed models or log binomial/identity binomial exchangeable generalised estimating equations. All models with repeated measures included the baseline score as the first time point.

Sensitivity and supportive analyses of amputation free survival, overall survival, and time to major amputation included a per protocol analysis based only on participants who were adherent. Preplanned subgroup analyses of amputation free survival were completed for the minimisation variables, with the exception of the recruiting centre. The effects of these subgroups were examined by adding the subgroup by treatment interaction parameters to the regression model. Subgroup specific hazard ratios and the ratio of hazard ratios were estimated from the model coefficients.

Multiple imputation was not used for any missing outcome data. Binary clinical outcome data were analysed in a time-to-event framework, censoring on last known follow-up. Other outcomes that were measured at several time points were analysed using repeated measures generalised linear mixed models with a compound symmetry covariance structure, which includes an implicit imputation of general missing data.

All analyses were done in SAS (version 9.4) or Stata (version 18.0). The trial steering committee provided independent oversight. Interim analyses of effectiveness and safety endpoints were performed on behalf of the data monitoring committee on an approximately annual basis during recruitment, using the Haybittle-Peto principle, so that no adjustment was made to the final P values. The trial was registered (ISRCTN14469736).

### Patient and public involvement

We have been supported throughout the trial by a designated patient and public involvement (PPI) group who were part of the trial steering committee. PPI members were consulted throughout the trial to improve our understanding of the needs of patients with chronic limb threatening ischaemia. PPI members commented on all patient facing material to ensure that they were clear and comprehensive. We organised collaboration days during the trial and the PPI representatives attended these meetings.

## Results

**Fig 1:**
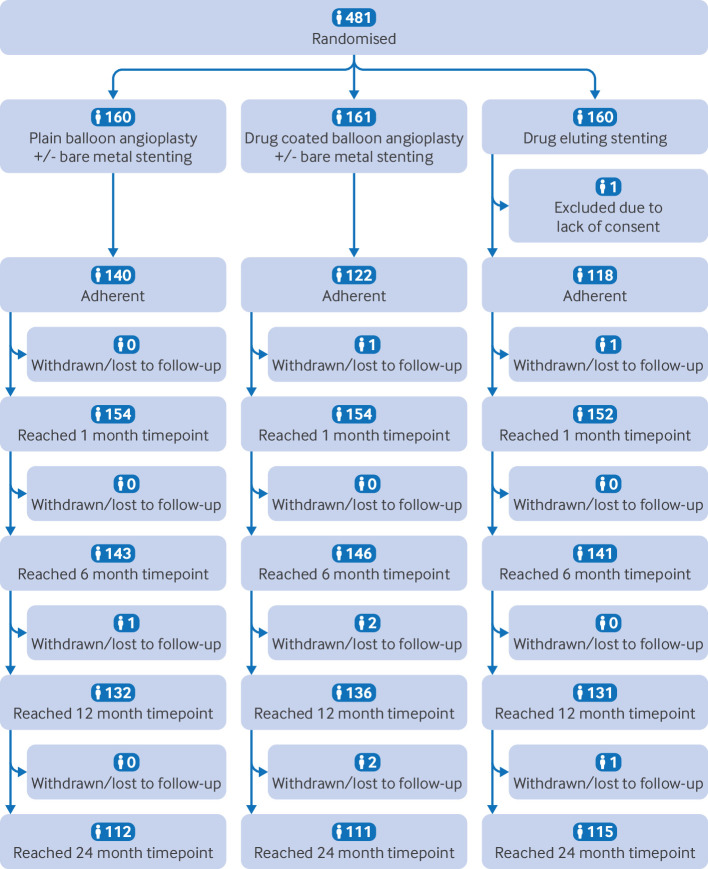
Consolidated standards of reporting trials (CONSORT) diagram. Assessment conducted refers to clinical review, not assessment of primary endpoint

Between 29 January 2016 and 31 August 2021, 481 participants were randomised to PBA±BMS (n=160), DCBA±BMS (n=161), or DES (n=160; fig 1). On 12 December 2018, recruitment was stopped by the trial management group, supported by the trial steering committee, because a meta-analysis had shown excess mortality in patients treated with a paclitaxel DCBA or DES.^12^ The UK Medicine and Healthcare products Regulatory Agency convened an expert advisory group, which concluded that paclitaxel devices could still be used in patients with chronic limb threatening ischaemia and recommended that BASIL-3 resume recruitment. With support from the funder and the trial steering committee, including PPI members, new ethical approval was obtained, and BASIL-3 reopened to recruitment on 16 September 2019 (table S1 gives more timeline details).

One participant was randomised to DES without written informed consent and so was removed from all analyses. Of the 480 participants included in the analyses, 167 (35%) were women, and the mean age was 71.8 years (standard deviation 10.8; table 1, table 2). A total of 464 (97%) participants received an endovascular procedure as their first revascularisation, with 444 (93%) of these being at least to the superficial femoral artery, the popliteal artery, or both. Three participants received a surgical revascularisation and 13 received no revascularisation. The allocated device was used in 142 (92%), 127 (82%), and 118 (76%) first endovascular interventions (in any femoro-popliteal artery) in the PBA±BMS, DCBA±BMS, and DES groups, respectively. This gave overall adherence rates of 140 (88%) for PBA±BMS, 122 (76%) for DCBA±BMS, and 118 (74%) for DES. A total of 426 (91%) participants received their first revascularisation within two weeks of randomisation and the median time to first intervention after randomisation was 0 days (interquartile range 0-3 days) in all three groups. Further details of the first revascularisation procedure including devices used and arterial segments treated can be found in the supplementary appendices (table S2). No patients were reported as having treatment using intravascular ultrasound as an adjunct, or with non-drug specialty balloons, or with atherectomy (such devices were not part of the protocol and were rarely used during the trial recruitment period in the UK).

**Table 1:** Baseline characteristics by randomised group

Characteristics	PBA±BMS (n=160)	DCBA±BMS (n=161)	DES (n=159)*
Age† (years)			
≤60	28 (18)	24 (15)	26 (16)
61-70	46 (29)	47 (29)	44 (28)
71-80	55 (34)	54 (34)	55 (35)
>80	31 (19)	36 (22)	34 (21)
Mean (SD)	71.5 (10.6)	72.1 (10.6)	71.7 (11.2)
Sex†			
Female	55 (34)	57 (35)	55 (35)
Male	105 (66)	104 (65)	104 (65)
Diabetes†			
Yes	89 (56)	88 (55)	87 (55)
No	71 (44)	73 (45)	72 (45)
Chronic kidney disease†‡			
Yes	53 (33)	55 (34)	54 (34)
No	107 (67)	106 (66)	105 (66)
Severity of clinical disease†			
Rest or night pain only	41 (26)	39 (24)	40 (25)
Tissue loss	31 (19)	31 (19)	27 (17)
Both	88 (55)	91 (57)	92 (58)
Artery intended for treatment†			
Superficial femoral only	102 (64)	104 (65)	101 (64)
Popliteal only	14 (9)	14 (9)	13 (8)
Both	44 (28)	43 (27)	45 (28)
Previous permissible intervention to the trial leg†			
Yes	23 (14)	28 (17)	29 (18)
No	137 (86)	133 (83)	130 (82)
Hybrid procedure planned†			
Yes	16 (10)	13 (8)	14 (9)
No	144 (90)	148 (92)	145 (91)
Trial leg			
Right	79 (49)	88 (55)	69 (43)
Left	81 (51)	73 (45)	90 (57)
Body mass index			
Mean (SD), number	27.6 (6.0), 147	27.6 (7.0), 142	27.0 (6.1), 148
Missing	13	19	11
eGFR (ml/min/1.73 m^2^)			
Mean (SD), number	65.9 (23.7), 160	66.6 (23.1), 161	64.8 (23.2), 159
Mobility			
Fully ambulant without walking aid	67 (42)	58 (36)	67 (42)
Ambulant with walking aid	80 (50)	84 (52)	81 (51)
Wheelchair bound	13 (8)	17 (11)	10 (6)
Bed bound	0 (–)	2 (1)	1 (1)
Smoking status			
Never	34 (21)	26 (16)	25 (16)
Former	91 (57)	86 (53)	91 (58)
Current	35 (22)	49 (30)	42 (27)
Missing	0	0	1
Pack years (former or current smokers)			
Mean (SD), number	36.0 (23.7), 105	39.6 (33.3), 114	37.1 (27.6), 110
Missing	21	21	23
Ethnicity			
White	151 (94)	151 (94)	149 (93)
Black/Black British	5 (3)	5 (3)	1 (1)
Asian/Asian British	3 (2)	4 (3)	7 (4)
Mixed	0 (–)	0 (–)	2 (1)
Chinese or other ethnic group	1 (1)	0 (–)	0 (–)
Missing	0	1	0
Medical history			
Stroke	24 (15)	20 (12)	25 (16)
Myocardial infarction	34 (21)	32 (20)	29 (18)
Missing	0	2	0
Angina	28 (18)	30 (19)	20 (13)
Missing	0	2	2
Coronary artery bypass graft	20 (13)	18 (11)	17 (11)
Percutaneous coronary intervention	18 (11)	16 (10)	17 (11)
Missing	1	1	2
Dialysis	7 (4)	5 (3)	10 (6)
Missing	0	0	1
Imaging method			
Duplex ultrasound only	58 (36)	50 (31)	62 (39)
MRA only	21 (13)	18 (11)	16 (10)
CTA only	43 (27)	54 (34)	39 (25)
DSA only	8 (5)	11 (7)	7 (4)
Duplex ultrasound and MRA	12 (8)	7 (4)	6 (4)
Duplex ultrasound and CTA	10 (6)	13 (8)	20 (13)
Duplex ultrasound and DSA	6 (4)	6 (4)	8 (5)
CTA and DSA	1 (1)	1 (1)	0 (–)
Missing	1	1	1

Data are numbers (%) unless stated otherwise.

BMS=bare metal stent; CTA=computed tomography angiography; DCBA=drug coated balloon angioplasty; DES=drug eluting stent; DSA=digital subtraction angiography; eGFR=estimated globular filtration rate; MRA=magnetic resonance angiography; PBA=plain balloon angioplasty; SD=standard deviation.

* Excluding participant who did not provide consent.

† Minimisation variables.

‡ Chronic kidney disease defined as stage 3 or worse based on eGFR<60 (ml/min/1.73 m^2^).

**Table 2:** Medical treatment by randomised group

Medical treatment	PBA±BMS (n=160)	DCBA±BMS (n=161)	DES (n=159)*
Antiplatelets and anticoagulants			
Both	18 (11)	20 (12)	24 (15)
Only antiplatelet	100 (63)	98 (61)	97 (61)
Only anticoagulant	20 (13)	22 (14)	20 (13)
Nether antiplatelet nor anticoagulant	22 (14)	21 (13)	18 (11)
Aspirin	77 (49)	67 (42)	81 (51)
Missing	2	0	0
Clopidogrel	55 (35)	61 (38)	51 (32)
Missing	3	0	0
Warfarin	18 (11)	12 (8)	17 (11)
Missing	2	3	0
Any other antiplatelet or anticoagulant	30 (19)	38 (25)	32 (21)
Missing	5	6	4
Analgesics			
Any analgesic	119 (76)	133 (85)	128 (82)
Missing	3	4	2
Paracetamol	98 (62)	110 (69)	102 (65)
Missing	1	1	1
Opiates	60 (38)	87 (55)	80 (51)
Missing	3	2	1
Non-steroidal anti-inflammatory drugs	9 (6)	12 (8)	14 (9)
Missing	1	7	2
Gabapentin or pregabalin	30 (20)	32 (21)	33 (22)
Missing	8	9	7
Amitriptyline	19 (12)	21 (13)	22 (14)
Missing	2	2	1
Other medical treatment			
Treatment for hypercholesterolemia	124 (79)	121 (75)	119 (75)
Missing	4	0	0
Treatment for hypertension	116 (76)	124 (78)	113 (72)
Missing	7	1	2
Previous vascular intervention to the trial leg†			
Endovascular	19 (12)	23 (14)	21 (13)
Surgery	6 (4)	12 (7)	10 (6)
Minor amputation	7 (4)	14 (9)	10 (6)
Missing	1	0	0
Previous vascular intervention to the non-trial leg†			
Endovascular	31 (19)	27 (17)	23 (14)
Surgery	13 (8)	17 (11)	13 (8)
Minor amputation	8 (5)	11 (7)	8 (5)
Below knee amputation	5 (3)	7 (4)	3 (2)
Above knee amputation	3 (2)	5 (3)	5 (3)
Missing	0	1	0

Data are numbers (%).

BMS=bare metal stent; DCBA=drug coated balloon angioplasty; DES=drug eluting stent; PBA=plain balloon angioplasty.

* Excluding participant who did not provide consent.

† Not mutually exclusive.

**Fig 2:**
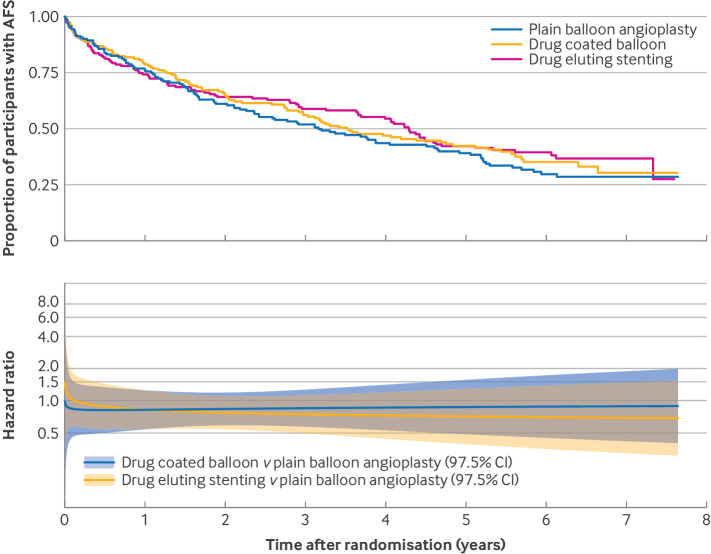
Amputation free survival (AFS) Kaplan-Meier plot and hazard ratio over time fitted assuming non-proportional hazards (intention-to-treat analysis). CI=confidence interval

The median time to last clinical follow-up was 2.1 years (range 0-7.2 years) for all participants, and 3.1 years (range 0-7.2 years) in survivors. In the PBA±BMS group, 106/160 (66%) participants had a major amputation or died (no amputation free survival) compared with 97/161 (60%) in the DCBA±BMS group (adjusted hazard ratio from the Cox proportional hazards model 0.84; 97.5% confidence interval 0.61 to 1.16; P=0.22), and 93/159 (58%) in the DES group (0.83, 0.60 to 1.15; P=0.20; table 3, fig 2). The median amputation free survival time was 3.16, 3.52, and 4.29 years in the PBA±BMS, DCBA±BMS, and DES groups, respectively. The absolute differences in failure probabilities and corresponding numbers needed to treat were −0.042 (97.5% confidence interval −0.164 to 0.079), NNTB 24 (NNTB 6 to ∞ to NNTH 13) and −0.031 (−0.152 to 0.091), NNTB 32 (NNTB 7 to ∞ to NNTH 11) between DCBA±BMS and PBA±BMS, and between DES and PBA±BMS, respectively, at two years (table S5). The per protocol sensitivity analysis produced consistent results (table 3). Model assumption checks were performed to assess the non-proportional hazard assumption, which was found to be violated (fig 2). Flexible parametric models were fitted and figure 2 presents a plot of the hazard ratios over time with 97.5% confidence intervals. There was no evidence of varying effects in the prespecified subgroup analyses (tables S3 and S4).

**Table 3:** Primary and secondary clinical outcomes

Outcomes	PBA±BMS (n=160)	DCBA±BMS (n=161)	DES (n=159)*	DCBA *v* PBA, estimate (97.5% CI)	DES *v* PBA, estimate (97.5% CI)
**Primary outcome**					
No amputation free survival (intention to treat)	106 (66)	97 (60)	93 (58)	HR 0.84 (0.61 to 1.16),† P=0.22	HR 0.83 (0.60 to 1.15),‡ P=0.20
No amputation free survival (per protocol)	91/140 (65)	74/122 (61)	71/118 (60)	HR 0.85 (0.59 to 1.22)†	HR 0.89 (0.62 to 1.28)‡
**Secondary outcomes**					
Death from any cause (intention to treat)	96 (60)	90 (56)	80 (50)	HR 0.86 (0.62 to 1.20)†	HR 0.79 (0.56 to 1.11)‡
Death from any cause (per protocol)	83/140 (59)	69/122 (57)	58/118 (49)	HR 0.87 (0.60 to 1.27)†	HR 0.78 (0.53 to 1.16)‡
Major amputation (intention to treat)	23 (14)	18 (11)	25 (16)	CHSR 0.74 (0.36 to 1.50)†; SHR 0.76 (0.37 to 1.53)§	CSHR 1.07 (0.56 to 2.05)‡; SHR 1.11 (0.58 to 2.11)¶
Major amputation (per protocol)	20/140 (14)	14/122 (11)	20/118 (17)	CHSR 0.76 (0.36 to 1.69)†; SHR 0.79 (0.35 to 1.76)§	CSHR 1.15 (0.56 to 2.37)‡; SHR 1.21 (0.59 to 2.48)¶
Further interventions	48 (30)	46 (29)	44 (28)	RR 0.96 (0.66 to 1.40)†; RD −0.003 (−0.113 to 0.107)**	RR 0.94 (0.64 to 1.37)‡; RD −0.032 (−0.148 to 0.084)††
Major adverse limb events	59 (37)	56 (35)	57 (36)	RR 0.92 (0.66 to 1.28)†; RD −0.015 (−0.129 to 0.099)**; CSHR 0.95 (0.62 to 1.44)†; SHR 0.99 (0.64 to 1.51)§	RR 0.98 (0.71 to 1.34)‡; RD −0.026 (−0.147 to 0.095)††; CSHR 0.94 (0.62 to 1.42)‡; SHR 0.96 (0.64 to 1.46)¶
Major adverse cardiovascular events	49 (31)	63 (39)	61 (38)	RR 1.28 (0.90 to 1.80)†; RD 0.092 (−0.025 to 0.208)‡‡; CSHR 1.31 (0.85 to 2.02)†; SHR 1.44 (0.94 to 2.26)§	RR 1.29 (0.91 to 1.83)‡; RD 0.073 (−0.045 to 0.192)§§; CSHR 1.28 (0.83 to 1.97)†; SHR 1.35 (0.88 to 2.07)§
30 day morbidity	45 (28)	53 (33)	51 (32)	RR 1.18 (0.82 to 1.69)§; RD 0.048 (−0.067 to 0.163)¶¶	RR 1.16 (0.80 to 1.68)¶; RD 0.040 (−0.076 to 0.155)***
30 day mortality	7 (4)	3 (2)	3 (2)	RR 0.41 (0.09 to 1.97)§; RD −0.025 (−0.069 to 0.018)¶¶	RR 0.41 (0.08 to 2.12)¶; RD −0.025 (−0.069 to 0.019)***
Opiate usage					
Baseline	60/157 (38)	87/159 (55)	80/158 (51)	NA	NA
1 month	51/134 (38)	70/136 (51)	52/140 (37)	RR 1.32 (0.98 to 1.77)§; RD 0.129 (−0.001 to 0.259)‡‡	RR 0.96 (0.69 to 1.35)¶; RD −0.013 (−0.139 to 0.114)§§
12 months	42/108 (39)	39/115 (34)	41/114 (36)	RR 0.83 (0.57 to 1.21)§; RD −0.050 (−0.186 to 0.086)‡‡	RR 0.88 (0.61 to 1.28)¶; RD −0.024 (−0.165 to 0.116)§§
24 months	29/84 (35)	31/91 (34)	24/99 (24)	RR 0.91 (0.58 to 1.42)§; RD −0.008 (−0.156 to 0.140)‡‡	RR 0.65 (0.39 to 1.08)¶; RD −0.099 (−0.236 to 0.037)§§
PEDIS, mean (SD), number					
Baseline	10.09 (1.93), 77	10.36 (2.04), 74	10.27 (1.93), 78	NA	NA
1 month	9.29 (1.84), 48	10.04 (2.09), 54	9.44 (2.15), 52	MD 0.58 (−0.26 to 1.42)‡‡	MD −0.06 (−0.90 to 0.78)§§
12 months	9.50 (2.51), 6	9.67 (1.83), 12	7.95 (1.85), 20	MD −0.01 (−2.01 to 1.99)‡‡	MD −1.46 (−3.33 to 0.40)§§
24 months	7.50 (0.71), 2	9.67 (2.16), 6	8.50 (2.11), 12	MD 1.45 (−1.78 to 4.68)‡‡	MD 0.96 (−2.06 to 3.98)§§
WIFi					
Baseline	21/44 (48)	30/57 (53)	29/53 (55)	NA	NA
1 month	5/27 (19)	6/28 (21)	4/27 (15)	Not estimable	Not estimable
12 months	1/6 (17)	2/7 (29)	5/14 (36)	Not estimable	Not estimable
24 months	1/1 (100)	2/2 (100)	1/5 (20)	Not estimable	Not estimable
ABPI, mean (SD), number					
Baseline	0.45 (0.44), 48	0.46 (0.41), 59	0.51 (0.38), 44	NA	NA
1 month	0.64 (0.43), 28	0.72 (0.45), 28	0.66 (0.45), 18	MD 0.03 (−0.21 to 0.27)†††	MD −0.02 (−0.29 to 0.25)‡‡‡
12 months	0.57 (0.42), 19	0.45 (0.53), 11	0.61 (0.46), 10	MD 0.04 (−0.29 to 0.36)†††	MD 0.00 (−0.34 to 0.34)‡‡‡
24 months	0.70 (0.52), 13	0.63 (0.50), 12	0.72 (0.40), 10	MD 0.02 (−0.32 to 0.37)†††	MD 0.06 (−0.30 to 0.43)‡‡‡
TBPI, mean (SD), number					
Baseline	0.21 (0.32), 9	0.27 (0.41), 17	0.20 (0.32), 13	NA	NA
1 month	0.21 (0.28), 6	0.31 (0.54), 3	0.50 (0.15), 5	MD 0.18 (−0.09 to 0.46)†††	MD 0.17 (−0.08 to 0.41)‡‡‡
12 months	0.45 (0.24), 2	0.26 (0.36), 2	0.94 (–), 1	MD 0.75 (0.45 to 1.05)†††	MD 0.04 (−0.22 to 0.31)‡‡‡
24 months	1.27 (–), 1	—§§§	—§§§	Not estimable	Not estimable
Serious adverse events	16 (10)	9 (6)	17 (11)	RR 0.53 (0.22 to 1.30)† RD −0.044 (−0.111 to 0.023)¶¶	RR 0.98 (0.48 to 2.00)† RD 0.007 (−0.070 to 0.083)***

Data are numbers (%) unless stated otherwise.

ABPI=ankle brachial pressure index; BMS=bare metal stent; CI=confidence interval; CSHR=cause specific hazard ratio; DCBA=drug coated balloon angioplasty; DES=drug eluting stent; HR=hazard ratio; MD=mean difference; NA=not applicable; PBA=plain balloon angioplasty; PEDIS=perfusion, extent, depth, infection and sensation; RD=risk difference; RR=risk ratio; SD=standard deviation; SHR=subdistribution hazard ratio; TBPI=toe brachial pressure index; WIFI=wound, ischemia, and foot infection.

* Excluding participant who did not provide consent.

† Adjusted for age, sex, diabetes mellitus, chronic kidney disease, severity of clinical disease, previous intervention to trial leg, intention for a hybrid procedure, intended artery for treatment and centre. Values <1 favour DCBA.

‡ Adjusted for age, sex, diabetes mellitus, chronic kidney disease, severity of clinical disease, previous intervention to trial leg, intention for a hybrid procedure, intended artery for treatment and centre. Values <1 favour DES.

§ Adjusted for age, sex, diabetes mellitus, chronic kidney disease, severity of clinical disease, previous intervention to trial leg, intention for a hybrid procedure and intended artery for treatment. Values <1 favour DCBA.

¶ Adjusted for age, sex, diabetes mellitus, chronic kidney disease, severity of clinical disease, previous intervention to trial leg, intention for a hybrid procedure and intended artery for treatment. Values <1 favour DES.

** Adjusted for age, sex, diabetes mellitus, chronic kidney disease, severity of clinical disease, previous intervention to trial leg, intention for a hybrid procedure, intended artery for treatment and centre. Values <0 favour DCBA.

†† Adjusted for age, sex, diabetes mellitus, chronic kidney disease, severity of clinical disease, previous intervention to trial leg, intention for a hybrid procedure, intended artery for treatment and centre. Values <0 favour DES.

‡‡ Adjusted for age, sex, diabetes mellitus, chronic kidney disease, severity of clinical disease, previous intervention to trial leg, intention for a hybrid procedure and intended artery for treatment. Values <0 favour DCBA.

§§ Adjusted for age, sex, diabetes mellitus, chronic kidney disease, severity of clinical disease, previous intervention to trial leg, intention for a hybrid procedure and intended artery for treatment. Values <0 favour DES.

¶¶ Unadjusted. Values <0 favour DCBA.

*** Unadjusted. Values <0 favour DES.

††† Adjusted for age, sex, diabetes mellitus, chronic kidney disease, severity of clinical disease, previous intervention to trial leg, intention for a hybrid procedure, intended artery for treatment and centre. Values >0 favour DCBA.

‡‡‡ Adjusted for age, sex, diabetes mellitus, chronic kidney disease, severity of clinical disease, previous intervention to trial leg, intention for a hybrid procedure, intended artery for treatment and centre. Values >0 favour DES.

§§§ No data available at this time point.

In the PBA±BMS group, 96/160 (60%) participants died from any cause compared with 90/161 (56%) in the DCBA±BMS group (adjusted hazard ratio 0.86, 97.5% confidence interval 0.62 to 1.20) and 80/159 (50%) in the DES group (0.79, 0.56 to 1.11). Table S8 presents causes of death. Model assumption checks were performed to assess the non-proportional hazard assumption, which was found to be violated (fig S1). Flexible parametric models were fitted and figure S1 provides a plot of the hazard ratio over time with 97.5% confidence intervals.

In the PBA±BMS group, 23/160 (14%) participants had a major amputation compared with 18/161 (11%) participants in the DCBA±BMS group (adjusted cause specific hazard ratio 0.74, 97.5% confidence interval 0.36 to 1.50; subdistribution hazard ratio 0.76, 97.5% confidence interval 0.37 to 1.53), and 25/159 (16%) in the DES group (1.07, 0.56 to 2.05; 1.11, 0.58 to 2.11).

For overall survival and time to major amputation, the per protocol sensitivity analyses produced consistent results (table 3). No differences were observed between the treatment groups relating to further interventions, 30 day morbidity and death, major adverse limb events, major adverse cardiovascular events, relief of ischaemic pain, or health related quality of life (table 3, table 4, table S6, and figures S2-S5).

**Table 4:** Patient reported secondary outcomes (intention-to-treat population)

Outcome	PBA±BMS (n=160)	DCBA±BMS (n=161)	DES (n=159*)	DCBA *v* PBA, mean difference (97.5% CI)	DES *v* PBA, mean difference (97.5% CI)
**Visual analogue scale**					
Baseline	6.72 (2.58), 147	6.46 (2.82), 147	6.64 (2.71), 141	NA	NA
1 month	4.15 (3.12), 110	4.15 (3.12), 110	4.15 (3.12), 110	0.00 (−0.86 to 0.86)†	−0.49 (−1.33 to 0.36)‡
12 months	4.42 (3.07), 104	4.42 (3.07), 104	4.42 (3.07), 104	−0.62 (−1.58 to 0.34)†	−0.77 (−1.71 to 0.17)‡
24 months	3.69 (3.03), 111	3.69 (3.03), 111	3.69 (3.03), 111	−0.20 (−1.26 to 0.86)†	−1.05 (−2.11 to 0.00)‡
**VascuQoL composite total score**					
Baseline	2.82 (1.09), 141	2.97 (1.21), 130	2.84 (1.24), 134	NA	NA
1 month	4.09 (1.67), 106	4.09 (1.67), 106	4.09 (1.67), 106	0.12 (−0.31 to 0.54)§	0.02 (−0.39 to 0.43)¶
12 months	4.09 (1.70), 87	4.09 (1.70), 87	4.09 (1.70), 87	0.25 (−0.20 to 0.70)§	0.26 (−0.19 to 0.71)¶
24 months	4.19 (1.53), 103	4.19 (1.53), 103	4.19 (1.53), 103	−0.05 (−0.55 to 0.46)§	0.19 (−0.30 to 0.68)¶
**EQ-5D-5L health state score**					
Baseline	51.9 (20.2), 149	53.0 (22.6), 147	51.4 (22.0), 145	NA	NA
1 month	57.7 (23.5), 113	57.7 (23.5), 113	57.7 (23.5), 113	0.0 (−6.4 to 6.5)**	−2.4 (−8.7 to 4.0)††
12 months	56.2 (23.8), 104	56.2 (23.8), 104	56.2 (23.8), 104	3.5 (−3.6 to 10.6)**	0.6 (−6.4 to 7.6)††
24 months	54.3 (22.5), 118	54.3 (22.5), 118	54.3 (22.5), 118	−3.1 (−11.0 to 4.8)**	2.7 (−5.0 to 10.4)††
**EQ-5D-5L index score**					
Baseline	0.254 (0.316), 147	0.298 (0.345), 146	0.279 (0.310), 144	NA	NA
1 month	0.457 (0.332), 113	0.457 (0.332), 113	0.457 (0.332), 113	−0.009 (−0.101 to 0.084)§	0.028 (−0.063 to 0.118)¶
12 months	0.410 (0.352), 105	0.410 (0.352), 105	0.410 (0.352), 105	−0.018 (−0.118 to 0.083)§	0.026 (−0.073 to 0.126)¶
24 months	0.474 (0.307), 115	0.474 (0.307), 115	0.474 (0.307), 115	−0.065 (−0.175 to 0.045)§	0.015 (−0.093 to 0.123)¶
**ICECAP-O**					
Baseline	0.695 (0.204), 139	0.689 (0.214), 140	0.672 (0.200), 137	NA	NA
1 month	0.688 (0.224), 106	0.688 (0.224), 106	0.688 (0.224), 106	0.048 (−0.011 to 0.107)§	0.031 (−0.028 to 0.089)¶
12 months	0.727 (0.202), 102	0.727 (0.202), 102	0.727 (0.202), 102	−0.001 (−0.064 to 0.063)§	−0.024 (−0.087 to 0.040)¶
24 months	0.711 (0.176), 108	0.711 (0.176), 108	0.711 (0.176), 108	−0.033 (−0.102 to 0.036)§	−0.009 (−0.077 to 0.059)¶
**SF-12 physical component score**					
Baseline	37.8 (5.9), 139	38.0 (5.5), 136	38.2 (6.6), 131	NA	NA
1 month	36.5 (6.9), 100	36.5 (6.9), 100	36.5 (6.9), 100	1.5 (−0.5 to 3.4)†	1.2 (−0.7 to 3.2)‡
12 months	38.0 (6.8), 95	38.0 (6.8), 95	38.0 (6.8), 95	−0.1 (−2.3 to 2.0)†	0.8 (−1.3 to 3.0)‡
24 months	37.8 (6.7), 108	37.8 (6.7), 108	37.8 (6.7), 108	−0.5 (−2.8 to 1.9)†	−0.9 (−3.2 to 1.5)‡
**SF-12 mental component score**					
Baseline	42.0 (9.0), 139	43.6 (9.5), 136	43.9 (9.2), 131	NA	NA
1 month	45.1 (9.4), 100	45.1 (9.4), 100	45.1 (9.4), 100	−1.3 (−4.1 to 1.4)§	−0.9 (−3.6 to 1.8)¶
12 months	43.8 (9.9), 95	43.8 (9.9), 95	43.8 (9.9), 95	−0.2 (−3.3 to 2.9)§	−1.3 (−4.4 to 1.7)¶
24 months	44.2 (9.2), 108	44.2 (9.2), 108	44.2 (9.2), 108	0.4 (−2.9 to 3.8)§	0.3 (−3.0 to 3.7)¶
**HADS anxiety subscale**					
Baseline	8.5 (5.0), 136	7.9 (4.8), 137	8.7 (5.1), 134	NA	NA
1 month	6.7 (4.4), 99	6.7 (4.4), 99	6.7 (4.4), 99	−0.0 (−1.4 to 1.4)†	0.7 (−0.7 to 2.0)‡
12 months	7.2 (5.0), 92	7.2 (5.0), 92	7.2 (5.0), 92	−0.1 (−1.5 to 1.4)†	0.6 (−0.9 to 2.1)‡
24 months	7.6 (4.9), 103	7.6 (4.9), 103	7.6 (4.9), 103	−0.2 (−1.8 to 1.4)†	−0.2 (−1.7 to 1.4)‡
**HADS depression subscale**					
Baseline	7.9 (4.3), 134	7.9 (4.3), 137	8.4 (4.5), 135	NA	NA
1 month	7.2 (4.6), 102	7.2 (4.6), 102	7.2 (4.6), 102	−0.1 (−1.4 to 1.2)†	0.8 (−0.5 to 2.1)‡
12 months	7.4 (5.1), 91	7.4 (5.1), 91	7.4 (5.1), 91	−0.6 (−2.0 to 0.8)†	0.5 (−0.9 to 1.9)‡
24 months	8.1 (4.8), 101	8.1 (4.8), 101	8.1 (4.8), 101	−0.2 (−1.7 to 1.4)†	−0.0 (−1.5 to 1.5)‡

Data are mean (standard deviation), number.

BMS=bare metal stent; CI=confidence interval; DCBA=drug coated balloon angioplasty; DES=drug eluting stent; EQ-5D-5L=Euroqol 5L; HADS=Hospital Anxiety and Depression Scale; ICDCAP-O=ICEpop capability measure for older people; NA=not applicable; PBA=plain balloon angioplasty; SF-12=Short Form 12; VascuQoL=Vascular Quality of Life Questionnaire.

* Excluding participant who did not provide consent.

† Adjusted for age, sex, diabetes mellitus, chronic kidney disease, severity of clinical disease, previous intervention to trial leg, intention for a hybrid procedure, intended artery for treatment and centre. Values <0 favour DCBA.

‡ Adjusted for age, sex, diabetes mellitus, chronic kidney disease, severity of clinical disease, previous intervention to trial leg, intention for a hybrid procedure, intended artery for treatment and centre. Values <0 favour DES.

§ Adjusted for age, sex, diabetes mellitus, chronic kidney disease, severity of clinical disease, previous intervention to trial leg, intention for a hybrid procedure and intended artery for treatment. Values <0 favour DCBA.

¶ Adjusted for age, sex, diabetes mellitus, chronic kidney disease, severity of clinical disease, previous intervention to trial leg, intention for a hybrid procedure and intended artery for treatment. Values <0 favour DES.

** Unadjusted. Values <0 favour DCBA.

†† Unadjusted. Values <0 favour DES.

In the PBA±BMS group, 16/160 (10%) participants had a serious adverse event compared with 9/161 (6%) in the DCBA±BMS group and 17/159 (11%) in the DES group. One serious adverse event was considered related to the trial intervention and was unexpected (hospital admission with epistaxis resolved with sphenopalatine artery ligation). Table S7 presents further serious adverse event details. Most causes of death were reported as multifactorial and often related to several comorbidities (table S8).

## Discussion

### Principal findings

The BASIL-3 trial showed that, in patients with chronic limb threatening ischaemia undergoing an endovascular femoro-popliteal with or without infra-popliteal revascularisation to restore limb perfusion, neither DCBA±BMS nor primary DES, when used in the femoro-popliteal segment, significantly improved amputation free survival when compared with PBA±BMS. The best estimates of the hazard ratios for amputation free survival were 0.84 for DCBA±BMS and 0.83 for DES, which is equivalent to a NNTB of 24 for DCBA±BMS and a NNTB of 32 for DES at the two year time point. The 97.5% confidence intervals for the hazard ratios ranged from 0.61 to 1.16 for DCBA±BMS and 0.60 to 1.15 for DES, which include values representing potential benefit and harm. The 97.5% confidence intervals narrowly excluded (or bordered) the 40% relative reduction (13% absolute difference at two years) in the rate of major amputation or death, which was set as the target difference a priori. Therefore, the BASIL-3 trial does not support the hypothesis that the use of DCBA±BMS, or DES, in the femoro-popliteal segment for revascularisation in patients with chronic limb threatening ischaemia confers important clinical benefit over PBA±BMS in terms of amputation free survival (the primary outcome) or a wide range of prespecified secondary clinical and patient reported outcomes. We cannot conclude that DCBA±BMS, or DES, do not potentially offer smaller clinical benefits over PBA±BMS, which some clinicians might consider meaningful, and BASIL-3 was not powered to detect.

### Strengths and limitations

BASIL-3 is a publicly funded randomised controlled trial evaluating DCBA±BMS and DES separately in patients with chronic limb threatening ischaemia. The number of primary endpoints required to attain at least 90% power to detect the prespecified target differences was exceeded. Follow-up was longer and more complete than anticipated. Cause of death data were available for all patients. BASIL-3 contains a within trial health economic analysis that will be reported separately. The results of the intention-to-treat and per protocol analyses were consistent, which highlights the robustness of the trial findings. BASIL-3 is a pragmatic, real world trial that involved most (35) of the major UK vascular units and so reflects standard of care across the NHS. The results are likely to be applicable to other countries with similar chronic limb threatening ischaemia populations and healthcare systems.

Because of concerns about the safety of paclitaxel, BASIL-3 recruitment was paused between December 2018 and September 2019.^12^ ^13^ ^14^ ^15^ ^16^ ^17^ ^18^ Although we have no evidence to suggest that this was the case, we cannot exclude the possibility that patients recruited before and after the pause were different. When covid-19 arrived in the UK in March 2020, recruitment and follow-up became increasingly difficult. However, we have no evidence to suggest that covid altered the outcome of the trial in terms of the differences observed between the three arms. Covid was recorded as the cause of death for only 12 patients.

In keeping with almost all other studies, BASIL-3 treats DCB and DES as classes of devices. Although it has been suggested that there might be clinically important differences between different types (brands) of DCB and DES, comparative data are virtually non-existent in chronic limb threatening ischaemia. The use of newer devices that became available during the BASIL-3 recruitment period was low. In terms of bailout stenting after PBA or DCB, biomimetic stenting was used in 13 patients in total from both cohorts. Similarly, the use of non-paclitaxel DCB (one patient, sirolimius) and non-paclitaxel DES (eight patients, everolimus) was exceedingly low. It is unknown whether these technologies are superior to standard BMS or paclitaxel based devices and the numbers of such in BASIL-3 are too small to make any meaningful comparisons. Details about vessel preparation were not collected or part of the protocol (such as atherectomy, intravascular ultrasound, intravascular lithotripsy etc). The use of such devices in the UK was low during the BASIL-3 recruitment period (most were recruited before 2020). It is unclear what additional benefit, if any, such devices provide in the absence of high quality, publicly funded evidence.

The non-adherence rate, especially in the drug eluting arms, was slightly higher than expected and the reasons for this are not completely clear. However, there were some instances where endovascular treatment was not possible. BASIL-3 was designed to be a pragmatic trial that is a true reflection of real world clinical practice. In the trial, 464/480 participants received an endovascular procedure as their first intervention. This was mostly driven by different devices being used at the discretion of the treating physician. Patients with a previous intervention (in the preceding 12 months) were excluded because it was felt that this would increase the risk of treatment failure with subsequent intervention. Also, given previous failed intervention, it was felt that clinicians might not have equipoise to try PBA again and would be more likely to use drug eluting technology or consider a different approach, which could have been a major barrier to reaching clinical equipoise.

### Comparison with other studies

Before BASIL-3, there was quantitatively and qualitatively limited and conflicting evidence regarding the clinical, and even more so the cost effectiveness of DCBA or DES in patients presenting with chronic limb threatening ischaemia.^19^ ^20^ ^21^ ^22^ Unlike BASIL-3, many studies analysed DCBA and DES together, even though they are very different technologies with their own advantages and disadvantages in different clinical and anatomic scenarios. As a result, there is considerable ongoing debate and controversy around the use of these devices in patients with chronic limb threatening ischaemia, and large variations remain in practice within and between countries.^23^ ^24^

In the UK, in 2012, the lack of evidence of clinical and cost effectiveness led NICE not to recommend DCBA or DES for the treatment of chronic limb threatening ischaemia. However, BASIL-3 was funded by NIHR HTA as a direct result of a NICE research recommendation and the trial results will be available to NICE when it comes to review its UK national guidelines on the management of chronic limb threatening ischaemia. BASIL-3 is likely to help inform vascular practice in other countries with similar chronic limb threatening ischaemia populations and healthcare systems.

BASIL-3 comprised an anatomically heterogeneous cohort typical of patients with chronic limb threatening ischaemia. We are collecting prerandomisation imaging to explore whether there are anatomic subgroups within the BASIL-3 cohort where DCBA or DES might confer clinical benefit. BASIL-3 did not include standardised follow-up imaging. At present, we do not have data on anatomic endpoints such as restenosis. However, a more detailed analysis of the nature and timing of further repeat and crossover interventions will be the subject of a further report. The BASIL-3 within-trial health economic analysis will be reported separately in due course.

### Policy implications

In the BASIL-3 trial the use of DCBA±BMS and DES in participants with chronic limb threatening ischaemia secondary to femoro-popliteal with or without infra-popliteal disease did not confer clinical benefit over the use of PBA±BMS. Therefore, in this patient group, BASIL-3 does not support a role for these technologies in terms of clinical effectiveness, based on the effect size that the trial was powered to determine. We cannot exclude smaller absolute differences in the primary outcome, but it is unclear if any smaller absolute difference would be clinically meaningful. A separate health economics report will be published in due course to assess the cost effectiveness and cost utility analyses of these devices.

### Conclusions

In the BASIL-3 trial, the use of DCBA±BMS and DES did not confer important clinical benefit over PBA±BMS in the femoro-popliteal segment in patients with chronic limb threatening ischaemia undergoing endovascular femoro-popliteal, with or without infra-popliteal, revascularisation.

**What is already known on this topic**

In 2012, the UK National Institute for Health and Care Excellence (NICE) established that a randomised trial was needed to compare the clinical effectiveness of endovascular strategies in patients undergoing primary revascularisation for chronic limb threatening ischaemia
These strategies include femoro-popliteal plain balloon angioplasty with or without bare metal stenting, drug coated balloon angioplasty with or without bare metal stenting, and drug eluting stenting
NICE recommended that such a trial be performed, and this led the UK National Institute for Health and Care Research, Health Technology Assessment (NIHR HTA) programme to fund the BASIL-3 trial reported here

**What this study adds**

Recent systematic reviews and meta-analyses have confirmed BASIL-3 is the only publicly funded randomised controlled trial to compare the clinical effectiveness of these three endovascular strategies in patients undergoing revascularisation for chronic limb threatening ischaemia
In the BASIL-3 trial, the use of drug coated balloons and drug eluting stents in the femoro-popliteal segment did not confer significant clinical benefit over the use of plain balloons and bare metal stents
BASIL-3 does not support a role for drug coated balloons or drug eluting stents in the femoro-popliteal segment in the management of patients with chronic limb threatening ischaemia undergoing endovascular revascularisation

## Data Availability

Requests for data should be directed to the corresponding author. Participant level data will be made available within six months of publication. Requests will be assessed for scientific rigour before being granted. Data will be anonymised and securely transferred. A data sharing agreement might be required.

## Web extra

Extra material supplied by authors

Web appendix: Supplementary appendices

## Notes

Contributors: AWB was the chief investigator and responsible for all aspects of study design, completion, and dissemination. CAM and JAH were responsible for the analysis of the trial data, verified the underlying data reported in the manuscript and coauthored this manuscript. MAP and LM recruited and followed up patients, collected, verified, and interpreted the data, and were members of the trial management and writing groups. MAP is also the submitting/corresponding author and should be considered as equal contribution as a second author. GRB helped design the protocol and case report forms, was a member of the trial management group, recruited and followed up patients, interpreted the data, and was a member of the writing group. LK helped design the case report forms, recruited and followed up patients, collected, verified, and interpreted the data, and was a member of the writing group. JJD helped design the study, interpreted the data, and was a member of the writing group. We confirm that all authors had full access to all the data in the study and had final responsibility for the decision to submit for publication. The corresponding author attests that all listed authors meet authorship criteria and that no others meeting the criteria have been omitted. AWB is guarantor.

## Notes

Transparency: The lead author (the manuscript’s guarantor) affirms that the manuscript is an honest, accurate, and transparent account of the study being reported; that no important aspects of the study have been omitted; and that any discrepancies from the study as planned (and, if relevant, registered) have been explained.

Dissemination to participants and related patient and public communities: The BASIL-3 team will engage with patient and public involvement (PPI) members regarding the dissemination of the trial results. Future research groups, taking forward the research recommendations from this project, would benefit from engaging with PPI representatives.

Provenance and peer review: Not commissioned; externally peer reviewed.
