# Supplementary material for: Plain versus drug balloon and stenting in severe ischaemia of the leg (BASIL-3): open label, three arm, randomised, multicentre, phase 3 trial
Source: BMJ. 2025 Feb 24;388:e080881. doi: 10.1136/bmj-2024-080881 (PMC11848676; doi:10.1136/bmj-2024-080881)
Supplement: Supplementary file 1 — Web appendix: Supplementary appendices [file braa080881.ww.pdf]

SUPPLEMENTARY APPENDICES

Table S1: Detailed timeline of the key decisions regarding the BASIL-3 recruitment pause and restart

Date	Action/Decision
6th Dec 2018	BASIL-3 team were made aware of the Katsanos meta-analysis.
11th Dec 2018	Decision was made to pause recruitment by the Basil-3 TMG which was supported by TSC chair (Jonathan Michaels).
12th Dec 2018	BASIL-3 sites were contacted, and recruitment was officially paused.
8th Jan 2019	The DMC met to review the current unblinded BASIL-3 data. The DMC recommended to the TSC (via a letter of recommendation) that follow-up continue until a minimum of two years follow-up post-randomisation in each patient.
18th Jan 2019	The TSC met to discuss the contents of the DMC letter. The TSC agreed to keep recruitment to BASIL-3 paused.
12th Apr 2019	The TSC met again and supported the reopening of BASIL-3 recruitment if MHRA did not advise against.
4th Jun 2019	The expert advisory group (EAG) set up by the MHRA convened and concluded that paclitaxel devices can still be used in patients with CLTI. The EAG also specifically recommended that BASIL-3 reopen to recruitment.

4th Jun 2019	The HTA met with the BASIL-3 team and the chair of TSC and agreed that BASIL-3 should reopen pending ethical approval.
31st Jul 2019	The BASIL-3 protocol and patient facing documentation were revised and submitted as a substantial amendment to the Research Ethics Committee, which was approved on 6th August 2019, with the aim to resume recruitment in September 2019.
16th Sep 2019	BASIL-3 re-opened to recruitment.

Table S2: Details of first revascularisation procedure

	**PBA +/- BMS N = 160**	**DCB +/- BMS N = 161**	**DES N = 159***
Endovascular intervention received	154 (96%)	154 (96%)	156 (98%)
Technical success[1]	129/154 (84%)	127/154 (82%)	130/156 (83%)
**Segments treated – N**			
SFA only	71	58	68
Popliteal only	4	8	9
SFA and Popliteal	41	43	43
Crural only	2	5	1
SFA and Crural	14	12	12
Popliteal and Crural	3	2	1
SFA, Popliteal and Crural	16	18	21
Other segment	0	3	0
No segment data available	3	5	1
**Devices used - N**			
**SFA only**	**71**	**58**	**68**
PBA only	56	1	5
DES only	0	1	29
PBA and DES	1	1	23
DCBA only	1	32	2
PBA and DCB	1	13	0
DCBA and DES	0	1	0
BMS only	2	1	0
PBA and BMS	5	1	1
DES and BMS	0	0	2
PBA, DES and BMS	0	0	1
DCBA and BMS	0	2	0
PBA, DCBA and BMS	0	2	0
No Device Data	5	3	5
**Popliteal only**	**4**	**8**	**9**

	PBA +/- BMS<br>N = 160	DCB +/- BMS<br>N = 161	DES<br>N = 159*
PBA only	4	1	0
DES only	0	0	4
PBA and DES	0	0	2
DCBA only	0	2	1
PBA and DCBA	0	1	0
DCBA and BMS	0	3	0
No Device Data	0	1	2
**SFA and Popliteal**	**41**	**43**	**43**
PBA only	23	3	9
DES only	0	1	6
PBA and DES	1	0	19
DCBA only	0	19	0
PBA and DCB	0	10	0
DCBA and DES	0	2	0
PBA, DCBA and DES	0	1	1
PBA and BMS	15	0	3
DES and BMS	0	0	2
PBA, DES and BMS	0	0	2
DCBA and BMS	0	2	0
PBA, DCBA and BMS	1	3	0
DCBA, DES and BMS	1	0	0
No Device Data	0	2	1
**Crural Artery(s) only**	**2**	**5**	**1**
PBA only	2	2	1
DCBA only	0	2	0
PBA and DCBA	0	1	0
**SFA and Crural Artery(s)**	**14**	**12**	**12**
PBA only	9	0	2
PBA and DES	1	0	10

	PBA +/- BMS<br>N = 160	DCB +/- BMS<br>N = 161	DES<br>N = 159*
PBA and DCB	0	9	0
DCBA and DES	0	3	0
PBA and BMS	4	0	0
**Popliteal and Crural Artery(s)**	**3**	**2**	**1**
PBA only	3	0	1
PBA and DCB	0	2	0
**SFA, Popliteal and Crural Artery(s)**	**16**	**18**	**21**
PBA only	15	1	3
PBA and DES	0	0	15
DCBA only	0	3	0
PBA and DCBA	0	12	0
DES and BMS	0	0	1
PBA, DES and BMS	1	0	1
PBA, DCBA and BMS	0	1	0
No Device Data	0	1	1
**Other Segment Only**	**0**	**3**	**0**
DCBA only	0	1	0
BMS only	0	1	0
PBA and BMS	0	1	0
**No Segment Data**	**6**	**7**	**3**

Abbreviations: BMS=Bare Metal Stent, DCBA=Drug Coated Balloon Angioplasty, DES=Drug Eluting Stent, PBA=Plain Balloon Angioplasty, SFA=Superficial Femoral Artery.
*Excluding the participant who did not provide consent.
[1]Techincal success is defined as technical success in all segments treated. If missing for all segments, the assumption is that it was not a technical success.

Table S3: Subgroup analyses for amputation free-survival

	PBA +/- BMS N = 160	DCBA +/- BMS N = 161	DES N = 159*	DCBA vs PBA	
				Hazard Ratio[1] (97.5% CI)	Ratio of Ratios
**Age at randomisation (years)**					
≤60	17/28 (61%)	11/24 (46%)	9/26 (35%)	0.60 (0.25 to 1.44)	REF
61-70	21/46 (46%)	20/47 (43%)	23/44 (52%)	0.84 (0.42 to 1.71)	1.41 (0.46 to 4.35)
71-80	41/55 (75%)	40/54 (74%)	35/55 (64%)	1.10 (0.67 to 1.82)	1.84 (0.67 to 5.09)
>80	27/31 (87%)	26/36 (72%)	26/34 (76%)	0.66 (0.35 to 1.24)	1.11 (0.38 to 3.26)
**Sex**					
Female	39/55 (71%)	34/57 (60%)	32/55 (58%)	0.88 (0.52 to 1.50)	1.07 (0.55 to 2.09)
Male	67/105 (64%)	63/104 (61%)	61/104 (59%)	0.82 (0.55 to 1.22)	REF
**DM**					
Yes	59/89 (66%)	55/88 (63%)	51/87 (59%)	0.88 (0.57 to 1.25)	1.11 (0.58 to 2.11)
No	47/71 (66%)	42/73 (58%)	42/72 (58%)	0.79 (0.49 to 1.28)	REF
**CKD**					
Yes	43/53 (81%)	39/55 (71%)	41/54 (76%)	0.79 (0.48 to 1.32)	0.91 (0.47 to 1.75)
No	63/107 (59%)	58/106 (55%)	52/105 (50%)	0.87 (0.58 to 1.32)	REF
**Severity of clinical disease**					
Ischaemic rest/night pain only	19/41 (46%)	20/39 (51%)	16/40 (40%)	1.09 (0.53 to 2.24)	REF
Tissue loss only	20/31 (65%)	18/31 (58%)	16/27 (59%)	0.68 (0.32 to 1.44)	0.63 (0.22 to 1.77)
Both	67/88 (76%)	59/91 (65%)	61/92 (66%)	0.83 (0.55 to 1.24)	0.76 (0.33 to 1.75)
**Artery being treated**					
Superficial femoral	71/102 (70%)	57/104 (55%)	60/101 (59%)	0.71 (0.47 to 1.06)	0.66 (0.32 to 1.35)
Popliteal	7/14 (50%)	10/14 (71%)	9/13 (69%)	1.36 (0.45 to 4.13)	1.26 (0.36 to 4.46)
Both	28/44 (64%)	30/43 (70%)	24/45 (53%)	1.07 (0.59 to 1.94)	REF
**Previous permissible intervention to the trial leg**					
Yes	17/23 (74%)	20/28 (71%)	15/29 (52%)	0.79 (0.37 to 1.70)	0.94 (0.41 to 2.18)
No	89/137 (65%)	77/133 (58%)	78/130 (60%)	0.84 (0.59 to 1.20)	REF
**Hybrid procedure planned**					
Yes	7/16 (44%)	6/13 (46%)	6/14 (43%)	1.29 (0.37 to 4.57)	1.59 (0.43 to 5.86)
No	99/144 (69%)	91/148 (61%)	87/145 (60%)	0.82 (0.57 to 1.13)	REF

Abbreviations: AFS=Amputation Free Survival, BMS=Bare Metal Stent, CI=Confidence Interval, CKD=Chronic Kidney Disease, DCBA=Drug Coated Balloon Angioplasty, DES=Drug Eluting Stent, DM=Diabetes Mellitus, PBA=Plain Balloon Angioplasty, REF=Reference category.
*Excluding the participant who did not provide consent.
[1]Adjusted for age, sex, DM, CKD, severity of clinical disease, previous intervention to the trial leg, intention for a hybrid procedure, intended artery for treatment and centre. Values <1 favour DCBA.

Table S4: Subgroup analyses for amputation free-survival

	PBA +/- BMS<br>N = 160	DCBA +/- BMS<br>N = 161	DES<br>N = 159*	DES vs PBA	
				Hazard Ratio[1]<br>(97.5% CI)	Ratio of Ratios
**Age at randomisation (years)**					
≤60	17/28 (61%)	11/24 (46%)	9/26 (35%)	0.44 (0.17 to 1.10)	REF
61-70	21/46 (46%)	20/47 (43%)	23/44 (52%)	1.04 (0.52 to 2.06)	2.38 (0.74 to 7.54)
71-80	41/55 (75%)	40/54 (74%)	35/55 (64%)	0.94 (0.56 to 1.58)	2.15 (0.74 to 6.22)
>80	27/31 (87%)	26/36 (72%)	26/34 (76%)	0.78 (0.41 to 1.46)	1.78 (0.58 to 5.46)
**Sex**					
Female	39/55 (71%)	34/57 (60%)	32/55 (58%)	0.78 (0.46 to 1.35)	0.91 (0.46 to 1.80)
Male	67/105 (64%)	63/104 (61%)	61/104 (59%)	0.86 (0.58 to 1.29)	REF
**DM**					
Yes	59/89 (66%)	55/88 (63%)	51/87 (59%)	0.87 (0.56 to 1.34)	1.11 (0.57 to 2.14)
No	47/71 (66%)	42/73 (58%)	42/72 (58%)	0.79 (0.48 to 1.28)	REF
**CKD**					
Yes	43/53 (81%)	39/55 (71%)	41/54 (76%)	0.86 (0.52 to 1.41)	1.05 (0.54 to 2.03)
No	63/107 (59%)	58/106 (55%)	52/105 (50%)	0.82 (0.53 to 1.25)	REF
**Severity of clinical disease**					
Ischaemic rest/night pain only	19/41 (46%)	20/39 (51%)	16/40 (40%)	0.78 (0.36 to 1.68)	REF
Tissue loss only	20/31 (65%)	18/31 (58%)	16/27 (59%)	0.75 (0.35 to 1.62)	0.97 (0.33 to 2.86)
Both	67/88 (76%)	59/91 (65%)	61/92 (66%)	0.87 (0.59 to 1.31)	1.13 (0.47 to 2.69)
**Artery being treated**					
Superficial femoral	71/102 (70%)	57/104 (55%)	60/101 (59%)	0.89 (0.60 to 1.32)	1.36 (0.63 to 2.93)
Popliteal	7/14 (50%)	10/14 (71%)	9/13 (69%)	1.22 (0.39 to 3.81)	1.87 (0.50 to 7.00)
Both	28/44 (64%)	30/43 (70%)	24/45 (53%)	0.65 (0.34 to 1.24)	REF
**Previous permissible intervention to the trial leg**					
Yes	17/23 (74%)	20/28 (71%)	15/29 (52%)	0.53 (0.23 to 1.21)	0.58 (0.27 to 1.43)
No	89/137 (65%)	77/133 (58%)	78/130 (60%)	0.91 (0.64 to 1.30)	REF
**Hybrid procedure planned**					
Yes	7/16 (44%)	6/13 (46%)	6/14 (43%)	1.12 (0.32 to 3.98)	1.38 (0.37 to 5.10)
No	99/144 (69%)	91/148 (61%)	87/145 (60%)	0.81 (0.58 to 1.14)	REF

Abbreviations: AFS=Amputation Free Survival, BMS=Bare Metal Stent, CI=Confidence Interval, CKD=Chronic Kidney Disease, DCBA=Drug Coated Balloon Angioplasty, DES=Drug Eluting Stent, DM=Diabetes Mellitus, PBA=Plain Balloon Angioplasty, REF=Reference category.
*Excluding the participant who did not provide consent.
[1]Adjusted for age, sex, DM, CKD, severity of clinical disease, previous intervention to the trial leg, intention for a hybrid procedure, intended artery for treatment and centre. Values <1 favour DES.

Table S5: Absolute differences in cumulative probabilities of death or major amputation and numbers need to treat by treatment group

**Time (years)**	**PBA +/- BMS** Cumulative probabilities (97.5% CI)	**DCBA +/- BMS** Cumulative probabilities (97.5% CI)	**DES** Cumulative probabilities (97.5% CI)	**DBCA vs PBA** Difference (97.5% CI) NNT (97.5% CI)	**DES vs PBA** Difference (97.5% CI) NNT (97.5% CI)
2	0.389 (0.309 to 0.482)	0.347 (0.270 to 0.439)	0.358 (0.280 to 0.451)	-0.042 (-0.164 to 0.079) NNTB 24 (NNTB 6 to ∞ to NNTH 13)	-0.031 (-0.152 to 0.091) NNTB 32 (NNTB 7 to ∞ to NNTH 11)
5	0.609 (0.521 to 0.698)	0.579 (0.490 to 0.103)	0.577 (0.487 to 0.670)	-0.031 (-0.158 to 0.097) NNTB 33 (NNTB 6 to ∞ to NNTH 10)	-0.032 (-0.160 to 0.097) NNTB 31 (NNTB 6 to ∞ to NNTH 10)

Abbreviations: BMS=Bare Metal Stent, CI=Confidence Interval, DCBA=Drug Coated Balloon Angioplasty, DES=Drug Eluting Stent, NNT=Number Needed to Treat, NNTB= Number Needed to Treat to Benefit, NNTH=Number Needed to Treat to Harm, PBA=Plain Balloon Angioplasty.

Table S6: Details of further interventions

Further Interventions	PBA +/- BMS N = 160	DCBA +/- BMS N = 161	DES N = 159*
**Number of participants**	48 (30%)	46 (29%)	44 (28%)
Any further Endovascular	37 (23%)	37 (23%)	29 (18%)
Any further Surgical Bypass	20 (13%)	14 (9%)	21 (13%)
Any further Non-bypass Surgery	4 (3%)	8 (5%)	5 (3%)
**Number of further interventions**	**77**	**69**	**60**
Endovascular	48	42	32
Surgical Bypass	23	14	23
Non-Bypass Surgery	4	8	5
Hybrid - Endovascular and Non-Bypass Surgery	1	3	0
Surgical and Non-Bypass Surgery	1	1	0
Hybrid - Endovascular, Bypass and Non-Bypass Surgery	0	1	0

Abbreviations: BMS=Bare Metal Stent, DCBA=Drug Coated Balloon Angioplasty, DES=Drug Eluting Stent, PBA=Plain Balloon Angioplasty.

Table S7: SAE details

Pat ID	Event description
**PBA +/- BMS**	
1	Hospitalisation. Patient admitted to ward after 2 week check, left leg no better. Patient to undergo CTA of left leg to assess. Details of relevant medical history: Diabetes, Hypertension, Triple Heart Bypass 14 years ago, CKD Stage 4.
2	Hospitalisation (8 nights) for pain in foot of trial leg and trash foot.
3	Death.
4	Death from Multi-organ failure
5	Worsening foot necrosis secondary to reperfusion injury.
6	Hospitalisation for lower respiratory tract infection. CRP 130, very SOB, not able to talk due to SOB. Death.
7	Embolisation required thrombolysis then had haematuria which resolved.
8	Hospitalisation with Gram+ Ve coccus in blood culture (sepsis), secondary to ulcer on left leg IV + oral antibiotics.
9	Retroperitoneal haematuria, tenderness r flank Hb 60.
10	Right thigh pain, gangrene of right hallux and 5th toe, patient underwent 1st and 5th toes amputation and revascularisation of right leg.
11	Infected hallux R – non-healing - required R hallux amputation.
12	Patient returned to theatre for right AKA - prolonged hospital stay.
13	Respiratory failure secondary to aspiration pneumonia. Brief PEA cardiac arrest, required ventilation and tracheostomy.
14	Hospitalisation with a right lump in the right groin. Swollen right leg. Had a right fem-pop bypass graft.
15	Failed angioseal-patient complained dof limb pain during recovery form initial angioplasty. Re-angio 4.5 cm proximal SFA occlusion - anticoagulated with Warfarin L/T.
16	Infected right heel ulcer.
**DCBA +/- BMS**	
17	Hospitalisation for wound infection.

Pat ID	Event description
18	Stent occlusion within 30 days.
19	Prolonged hospitalisation due to wound debridement, low Hb (61) and episodes of vomiting.
20	Lung infection requiring admission to hospital.
21	Prolonged hospitalisation for an amputation.
22	Ischemic right foot, CT angio confirmed blocked right SFT stent and lysis was required.
23	Infected non-healing wound.
	Coffee ground vomit with melaena, OGD done, duodenal ulcer, haemostatic clips applied.
24	Post procedure patient developed mechanical small bowel obstruction. Decompressed with NC tube 23lt drained at insertion. 5 day course of IU Abx for aspiration ap pneumonia. CT showed LT CFA aneurysm 2cm, had open repair of ltcfa pseudoaneurysm. Unwitnessed mechanical fall - no injuries sustained.
	*Hospitalisation with RT epitaxis. Polilateral rapid ohinos inserted. Bleeding continued despite this. Had emergency RT spheno palatine artery litigation. Bleeding stopped and discharged.
25	The patient suffered an acute stroke and died.
**DES**	
26	Death.
27	Death
28	Distal Embolization during procedure, rectified during procedure but precautionary o/n stay.
29	Haematoma on puncture site.
30	Vessel thrombosis during procedure -required thrombolysis.
31	Occulded stent.
32	Heavy growth of pseudomonas aerygiosa on right foot ulcer. Started IV abx.
33	Malpositioning of angioseal requiring surgical retrieval.
34	Pneumonia.
35	Bleeding from groin after SFA angioplasty. active extravasation from RCFA. Femoral angioplasty via left brachial access.
36	Hospital acquired pneumonia. Angioplasty r leg. Embolectomy required later for occlusion. Subsequent pyrexia and malaise. Aggressive antibiotic treatment was after some days eventually successful.
37	FAST atrial fibriliation post procedure treated with beta blockers and mgoxin.
38	Community acquired pneumonia, patient common artery pseudoaneurism.
39	Patient admitted with L left leg pain and numbness above knee. CT scan shows haematoma to left illiac muscle.
40	Rest Pain R forefoot. Re-admitted. Underwent right BKA then AKA.
41	One episode of spiked temperature having rigorously increased respiratory rate. CxR-no evidence of infection. Started on I/V tazocin and metronidazole for sepsis of there stepdown to after 48 hour to co-amoxiclav for 5/7. One episode of hypertension. Settled with amlodipine 5mg.
42	Admitted with left groin hematoma following vascular operation. Treated conservatively with I/VABX then to oral ABX. Duplex scan & CT scan shows no evidence of pseudoaneurysm. Patient had episodes of low blood sugar during admission.

Abbreviations: BMS=Bare Metal Stent, DCBA=Drug Coated Balloon Angioplasty, DES=Drug Eluting Stent, PBA=Plain Balloon Angioplasty, SAE=Serious Adverse Event, RUSAE=Related Unexpected Serious Adverse Event.
*RUSAE.

Table S8: Causes of death

Cause of death	PBA +/- BMS N = 96	DCBA +/- BMS N = 90	DES N = 80
Acute Limb Ischaemia	1	0	0
Chronic Limb Threatening Ischaemia	7	8	4
COVID	2	3	7
Cerebro-vascular Accident	6	5	5
Cardiac	26	37	16
Dementia	0	3	1
Frailty	0	0	1
Gastro-intestinal	2	2	3
Malignancy	15	9	13
Pulmonary Embolism	0	0	1
Ruptured Abdominal Aortic Aneurysm	0	1	0
Renal	2	3	2
Respiratory	19	14	16
Sepsis	7	2	3
Trauma	0	0	2
Multiple causes	4	1	3
Unknown	5	2	3

Abbreviations: BMS=Bare Metal Stent, DCBA=Drug Coated Balloon Angioplasty, DES=Drug Eluting Stent, PBA=Plain Balloon Angioplasty.

Table S9: The BASIL-3 Trial Sites & Prinicipal Investigators

**BASIL-3 sites in descending order of patients randomised**
University Hospital Birmingham (Heartlands Hospital): Dr Arul Ganeshan, Professor Andrew Bradbury [65]
Guy's & St Thomas' NHS FT (St Thomas' Hospital): Mr Hany Zayed [55]
University Hospitals of Leicester NHS Trust (Glenfield Hospital): Prof Athanasios Saratzis, Mr Robert Davies [43]
Hull University Teaching Hospitals NHS Trust: Professor Ian Chetter* [32]
Manchester University NHS FT (Wythenshawe Hospital): Dr Stephen Butterfield [31]
Sheffield Teaching Hospitals NHS FT (Northern General Hospital): Dr Stephen Goode [21]
Dorset County Hospital NHS FT: Mr James Metcalfe [20]
North Bristol NHS Trust (Southmead Hospital): Dr Peter Mezes [19]
The Dudley Group NHS FT (Russells Hall Hospital): Mr Simon Hobbs [18]
Aneurin Bevin University Health Board (Royal Gwent Hospital): Dr Nimit Goyal [16]
Leeds Teaching Hospitals NHS Trust: Dr Jai Patel* [13]
University Hospitals Sussex NHS FT (Royal Sussex Hospital): Mr Mario Caruana [13]
Manchester University NHS FT (Manchester Royal Infirmary): Mr Tawqeer Rashid [12]
East Kent Hospitals NHS FT (Kent & Canterbury Hospital): Dr Neelan Das [11]
United Lincolnshire Hospitals NHS Trust: Mr Nityanand Arya [11]
Frimley Health NHS FT (Frimley Park Hospital): Mr Patrick Chong [10]
Nottingham University Hospitals NHS Trust (Queens Medical Centre): Dr Said Habib [10]
Cardiff & Vale University Health Board (University Hospital Wales): Dr Richard White [9]
Northern Care Alliance NHS FT (Royal Oldham Hospital): Mr George Antoniou [8]
Imperial College Healthcare NHS Trust (St Marys Paddington): Professor Alun Davies* [7]
Royal Cornwall Hospital NHS Trust: Dr Angela Rogers [6]
Mid and South Essex NHS FT (Basildon University Hospital): Mr Shiva Dindyal [5]
NHS Forth Valley (Forth Valley Royal Hospital): Dr Nikolas Arestis [5]
NHS Lothian (Royal Infirmary Edinburgh): Dr Graham Weir [5]
County Durham & Darlington NHS FT: Mr Philip Davey [5]
St George's University Hospitals NHS FT: Dr Raj Das [5]
Newcastle upon Tyne Hospitals NHS FT (Freeman Hospital): Professor Gerard Stansby* [4]
North Cumbria Integrated NHS FT (Cumberland Infirmary): Mr Ron Eifell and Mr Philip Davey [4]
University Hospitals of North Midlands NHS Trust (Royal Stoke University Hospital): Mr John Asquith [4]
Royal Wolverhampton NHS Trust (New Cross Hospital): Mr Simon Hobbs [4]
University Hospital Dorset (Royal Bournemouth Hospital): Mr Lasantha Wijesinghe [3]
Bedford Hospital NHS Trust: Mr Arndam Chaudhuri [2]
East Suffolk & North Essex NHS FT (Colchester Hospital): Dr Nagendra Thayur and Mr James Metcalfe [2]
NHS Dumfries & Galloway (Dumfries & Galloway Royal Infirmary): Mr Joseph Sathianathan [2]

Royal Berkshire NHS FT (Royal Berkshire Hospital): Dr Farhan Ahmad [1]

**BASIL-3 Birmingham Clinical Trials Unit**
Suzanne Lockyer
Karim Jafri
Catherine Moakes
Jack Hall
Jonathan Deeks*
Hugh Jarrett*
Benjamin Hunt
Smitaa Patel*
Rebecca Record
Margaret Grant*
Gemma Slinn

*Co-applicants.

Figure S1: OS Kaplan Meier plot and hazard ratio over time fitted assuming non-proportional hazards (Intention to treat)

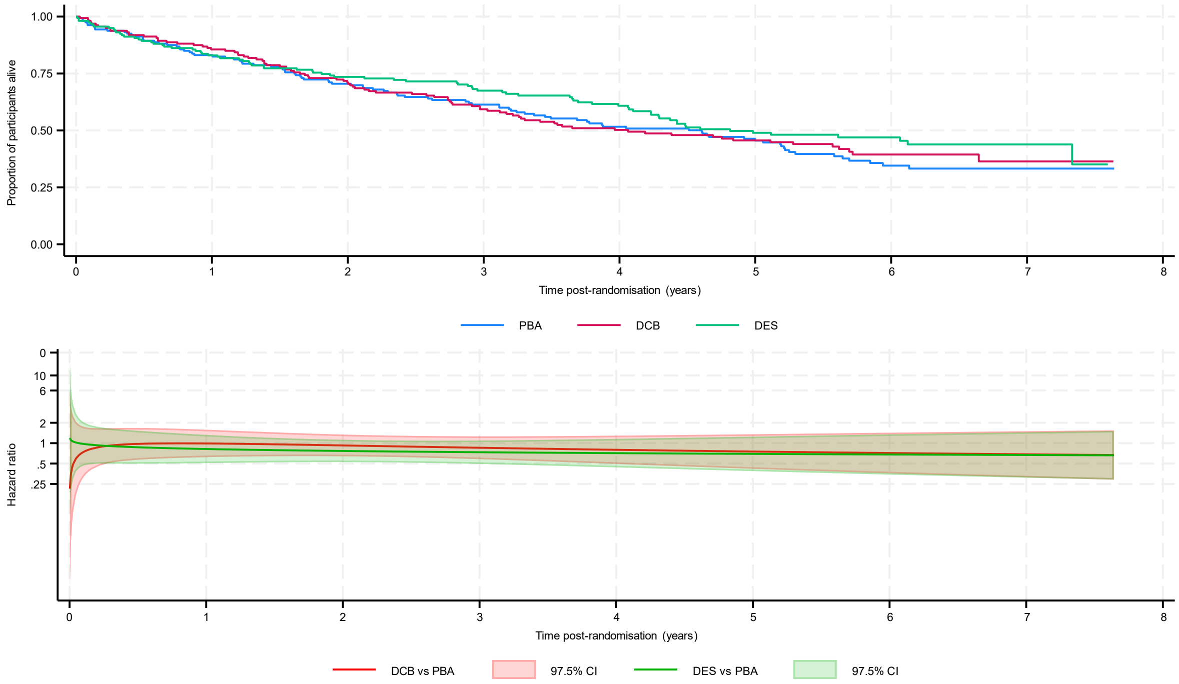

Figure S2: Further interventions plot

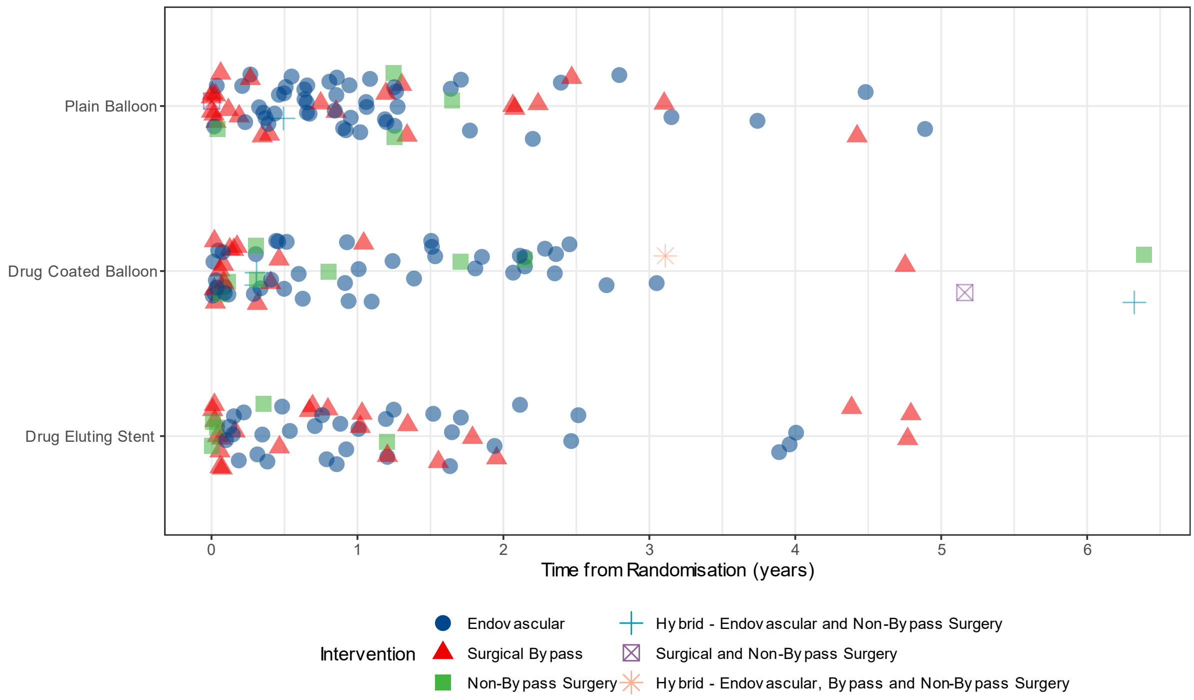

Figure S3: Cumulative incidence plot for time to major amputation

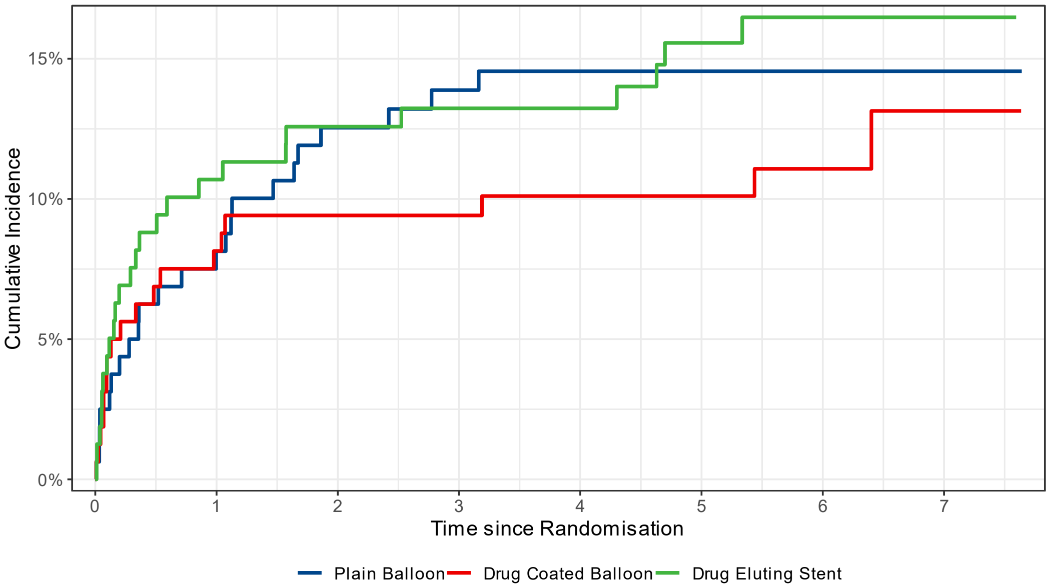

At Risk

	0	1	2	3	4	5	6	7
Plain Balloon	160	121	97	77	59	52	27	13
Drug Coated Balloon	161	124	102	81	62	55	20	5
Drug Eluting Stent	159	118	102	88	70	53	30	7

Figure S4: Cumulative incidence plot for time to Major Adverse Limb Events

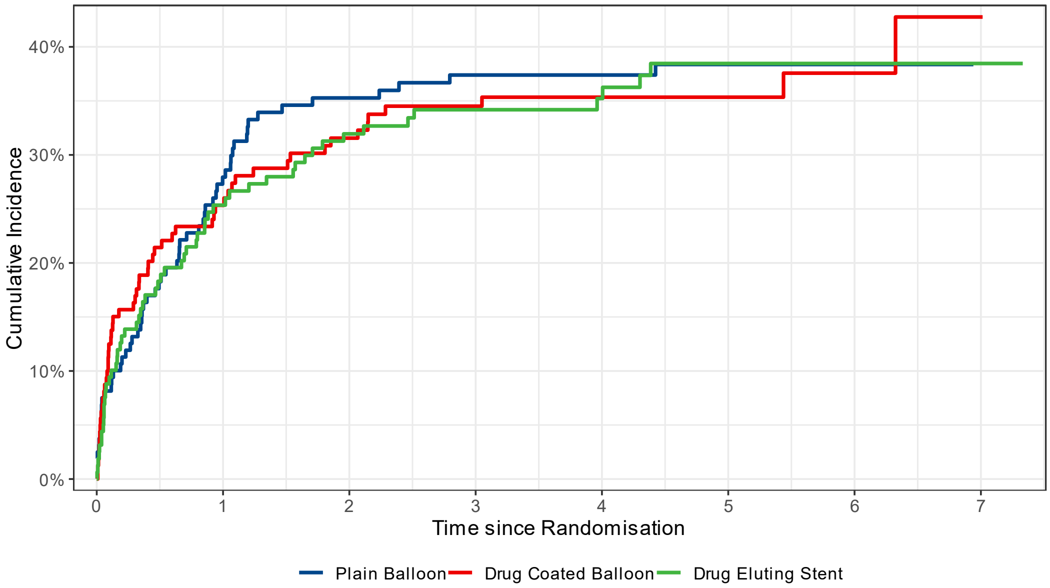

At Risk

Plain Balloon	160	89	66	44	29	22	2	0
Drug Coated Balloon	161	93	67	49	32	20	4	1
Drug Eluting Stent	159	93	71	52	35	13	7	1

Figure S5: Cumulative incidence plot for time to Major Adverse Cardiovascular Events

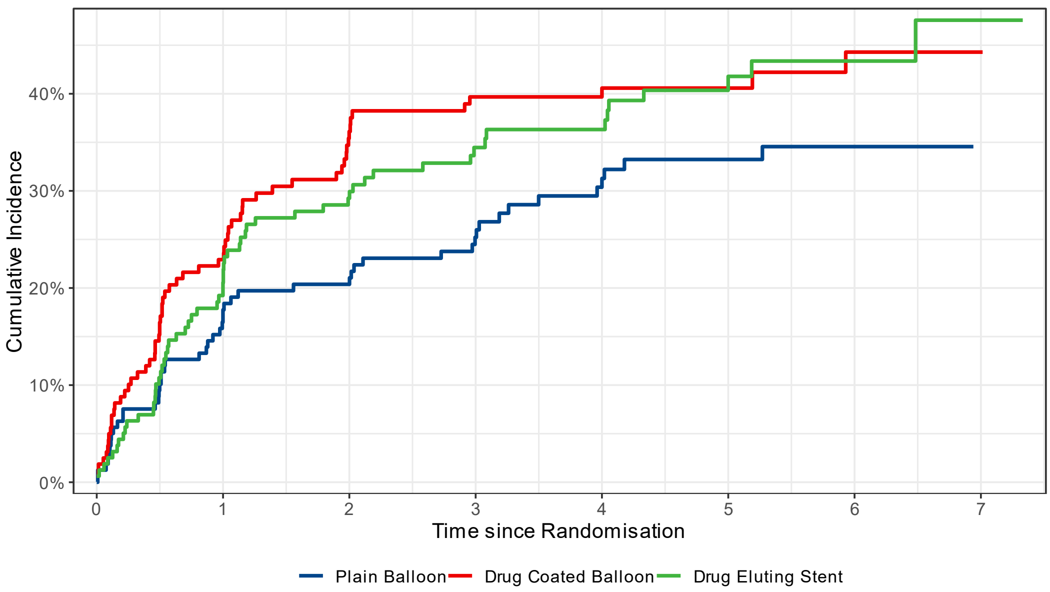

At Risk

Plain Balloon	160	105	84	59	35	20	2	0
Drug Coated Balloon	161	101	65	49	31	22	5	1
Drug Eluting Stent	159	99	73	49	33	11	5	2
